# Role of microvascular pericyte dysfunction in antibody-mediated rejection following kidney transplantation

**DOI:** 10.1080/0886022X.2025.2458749

**Published:** 2025-02-05

**Authors:** Jie Xu, Junyan Pu, Hao Chen, Li Sun, Shuang Fei, Zhijian Han, Jun Tao, Xiaobing Ju, Zijie Wang, Ruoyun Tan, Min Gu

**Affiliations:** a Department of Urology, The Second Affiliated Hospital of Nanjing Medical University, Nanjing, China; b Deparment of Urology, the First Affiliated Hospital of Nanjing Medical University, Nanjing, China; c The First Clinical Medical College, Nanjing Medical University, Nanjing, China

**Keywords:** Microvascular pericyte dysfunction, kidney transplantation, antibody-mediated rejection, endothelial cell damage, long-term prognosis

## Abstract

**Objective:**

To investigate the role of microvascular pericyte dysfunction in antibody-mediated rejection (ABMR) of transplanted kidneys.

**Methods:**

A total of 160 patients who underwent kidney transplantation in our hospital from 2004 to 2020 were enrolled, divided into 4 groups: ABMR group (*n* = 79), TCMR group (*n* = 20), mixed rejection group (*n* = 25) and control group (*n* = 36). Postoperative renal function indicators were compared, and immunohistochemical and immunofluorescence staining was performed on graft tissues and mice models using the pericyte marker PDGFR-β. An *in vitro* pericyte dysfunction model was co-cultured with vascular endothelial cells for functional assessment through Western blotting, PCR, and wound healing tests. KEGG pathway analysis from the GEO database identified gene expression changes in pericytes, which were further analyzed using electron microscopy and Western blot techniques.

**Results:**

There were statistically significant differences in creatinine, urea nitrogen, urine protein, and eGFR among the groups over time, with ABMR displaying the poorest outcomes. Immunohistochemistry revealed lower pericyte expression in ABMR, which was confirmed in mouse model studies showing reduced PDGFR-β expression in ABMR. KEGG analysis highlighted decreased autophagy in pericyte dysfunction, supported by electron microscopy and Western blot findings indicating reduced autophagy and pericyte damage, which could be reversed by chloroquine.

**Conclusion:**

ABMR episodes worsened the long-term prognosis of transplanted kidneys. pericyte dysfunction appears to be one of the crucial causes of poor prognosis in ABMR patients. *In vitro* studies demonstrated that dysfunction of microvascular pericytes can result in damage to vascular endothelial cells, with autophagy impairment being a significant mechanism contributing to pericyte dysfunction.

## Introduction

With advancements in kidney transplant surgery technology and the development of immunosuppression regimens, short-term survival rates for kidney transplants have significantly improved, while the incidence of T-cell-mediated acute rejection (TCMR) has decreased [1–3]. However, long-term graft survival rates remain low, mainly due to antibody-mediated rejection (ABMR) leading to late graft loss [4–5]. Challenges such as variable clinical symptoms of ABMR, limitations in antibody detection technology, and unstable C4d staining methods have hindered a full understanding of the immune damage mechanism of ABMR [6]. Current research suggests that ABMR affects vascular endothelial cells directly or indirectly, triggering the complement system or antibody-dependent cytotoxicity, initiating cellular and humoral immunity, and ultimately causing damage to the peritubular capillary basement membrane, arterial intimal fibrosis, and glomerular lesions in the transplanted kidney [7]. These effects may impact the long-term prognosis of transplanted kidneys, although the specific mechanism remains unclear. ABMR is a serious complication whose prognosis is influenced by several factors. If ABMR is not managed in a timely manner, it is likely to hasten the deterioration of transplanted kidney function and adversely impact the long-term outcomes of kidney transplantation. Furthermore, even when ABMR is effectively controlled, patients necessitate vigilant long-term monitoring to avert recurrence and additional immune complications [8]. Therefore, researchers are actively investigating the mechanism of renal vascular endothelial damage post-kidney transplantation in ABMR, and are continuously seeking strategies to reduce vascular endothelial damage and slow down the process of renal fibrosis, aiming to enhance the quality of kidney transplantation and improve long-term outcomes [9].

Pericytes, also known as Rouget cells or supporting cells, are found surrounding the endothelial cells of capillaries and venules in the body [10]. Embedded in the basement membrane, pericytes interact with endothelial cells through direct physical contact and paracrine signaling [11]. Together with endothelial cells, pericytes form the peritubular capillary (PTC) network in the kidney, which plays a crucial role in reabsorption, secretion, and oxygen delivery. Under normal conditions, pericytes stabilize blood vessel walls, while under pathological conditions, they can become activated, detach, and transform into myofibroblasts [12–14]. Damage to renal pericytes can lead to peritubular capillary (PTC) injury, where detached pericytes migrate away from capillaries into the interstitium, impacting the stability of blood vessels and contributing to renal fibrosis [15].

Numerous scholars have investigated the relationship between pericytes and endothelial cells, with research findings indicating a significant link between pericyte migration and endothelial cell instability. In cases where interactions between pericytes and endothelial cells are blocked, there is a prevention of microvascular rarefaction and interstitial fibrosis in renal injury. Pathologically, pericytes separate from endothelial cells and exhibit impaired function at the microvascular interstitial interface, leading to microvasculature instability and reduced blood vessel density, ultimately resulting in nephron ischemia [16].

Recent genetic fate mapping studies have revealed that pericytes are the primary source of scarring myofibroblasts in progressive chronic kidney disease [17]. Renal pericytes, characterized by platelet-derived growth factor receptor beta (PDGFR-β)(+)/neuroglial antigen 2(NG2)(+) expression, undergo abnormal transdifferentiation into myofibroblasts, marked by upregulation of α-SMA. The receptor tyrosine kinase PDGFR-β binds platelet-derived growth factor-β (PDGF-β) and plays a key role in activating pericytes, leading to their migration and recruitment to new blood vessel walls. NG2, a proteoglycan associated with pericytes during vascular morphogenesis, is involved in this process. Inhibition of vascular endothelial growth factor and platelet-derived growth factor can block this pathway [18–19].

Our previous studies have demonstrated the crucial role of renal microvascular endothelial-myofibroblast transdifferentiation and microvascular pericyte transdifferentiation in the development of interstitial fibrosis in transplanted kidneys [20]. DSA-mediated inflammatory injury to graft endothelial cells is a crucial mechanism underlying the development of antibody-mediated rejection (ABMR) following renal transplantation. Pericyte dysfunction can contribute to vascular endothelial injury, potentially exacerbating ABMR and resulting in more severe endothelial damage. Current research has yet to elucidate the causal relationship between these factors, indicating a need for further investigation. To investigate this, we conducted a study involving patients who underwent kidney transplantation at our center, adhering to specific criteria for inclusion and exclusion. We compared the postoperative renal function and outcomes among different patient groups, revealing that individuals with ABMR experienced a more pronounced decline in renal function post-transplantation. Pathological specimens from renal puncture and kidney sections from an ABMR mouse model were analyzed using various techniques such as chemical staining, immunofluorescence, and Western blotting to examine changes in peritubular capillaries and microvascular pericytes. Additionally, differences in the expression of markers associated with microvascular pericyte dysfunction were identified between the groups. Subsequently, an *in vitro* cell model with dysfunctional microvascular pericytes was established and co-cultured with vascular endothelial cells. The impact on vascular endothelial cells was assessed through Western blotting and PCR analyses. Functional alterations in vascular endothelial cells co-cultured with dysfunctional pericytes were investigated to explore the link between microvascular pericyte dysfunction and the prognosis of ABMR in transplanted kidneys. The mechanism of pericyte dysfunction in acute kidney injury was explored using data from the GEO database and KEGG analysis methods. The role of autophagy in pericyte dysfunction was further verified through transmission electron microscopy, Western blot, and other technologies.

## Materials and methods

### Study design and participants

We have conducted over 1,200 cases of related kidney transplantation and DCD donor kidney transplantation since 2004. We are currently following up with approximately 700 patients. All adult patients who received kidney transplants at the First Affiliated Hospital of Nanjing Medical University between July 2004 and September 2020 were initially included in this study, excluding those who had received other solid organ transplants or combined transplants. Clinical data was gathered from patients’ preoperative preparation and routine clinical follow-up, encompassing age, gender, surgery date, kidney donor source, antibody test outcomes, postoperative immunosuppressant regimen, serum creatinine levels, urea nitrogen levels, and urine protein test results.

All kidney transplant recipients underwent indication biopsy or protocol biopsy after transplantation, and the Banff (2019) pathological classification of allogeneic kidney transplantation was used for pathological classification and scoring. The transplanted kidney puncture biopsy is performed under ultrasound guidance. We avoid large blood vessels and randomly select locations at the lower pole of the kidney, puncturing two needles at each site to ensure that the collected tissue includes both cortex and medulla for subsequent tissue section. The study was approved by the Ethics Committee of The First Affiliated Hospital of Nanjing Medical University with written informed consent obtained from all subjects **(Trial Number: 2016-SR-029.A1)**.

The assessment of graft function relies on the estimated glomerular filtration rate (eGFR), which is calculated using the formula derived from the eGFR calculation method, as published by Stevens LA et al. in Kidney International in 2011 [21].

### Animals and experimental protocol

The ABMR model of mouse kidney transplantation was established as follows. Male C57BL/6 mice and Balb/c mice, aged 8-10 weeks and weighing approximately 25-30 g, were obtained from the Experimental Animal Center of Nanjing Medical University (License: SCXK (Su) 2021-0001). The mice were housed at the Experimental Animal Base of Nanjing Medical University (Emeiling) under controlled conditions (temperature: 20–26 °C, relative humidity: 40–60%, and 12h-12h light-dark cycle; License: SYXK (Su) 2018-0020). All animal procedures were approved by the Experimental Animal Welfare Ethics Committee of Nanjing Medical University (ethics number: IACUC-2109025). Full-thickness mouse skin transplantation was initially performed with C57BL/6 mice as recipients and Balb/c mice as donors.

The skin of C57BL/6 mice, measuring 1.5 cm × 1.5 cm, was harvested and transplanted onto the dorsal area of recipient Balb/c mice. Following 5 days of skin presensitization, a mouse kidney transplantation model was established. Anesthesia for the recipient Balb/c mice was induced using a small animal anesthesia machine with 3.5% isoflurane, followed by continuous inhalation of 1.5% isoflurane to maintain anesthesia. The donor left kidney was obtained and ectopically transplanted into the right side of the recipient using standard techniques. End-to-side anastomosis of the renal artery with Patch flap and anastomosis of the abdominal aorta with 10-0Prolene suture were performed as reported in literature. The renal vein was subjected to end-to-side anastomosis, while ureterovesical anastomosis was conducted using insertion method. Post-operation subcutaneous injection of 0.2 g/kg cefazoxime sodium (1 mL) was administered to prevent infection, and mice were housed individually with body temperature maintained using a constant temperature heating pad until sacrifice on day 5 for collection of peripheral blood and kidney tissue.

Subsequently, mice in the ABMR group underwent intraperitoneal heterotopic kidney transplantation 5 days post skin transplantation, with Balb/c mice as recipients and C57BL/6 mice as kidney donors. At 5 days post kidney transplantation, the recipients were euthanized under anesthesia (3% isoflurane) followed by cervical dislocation, and the transplanted kidney specimens were harvested. Each experimental group was *n* = 5 mice.

### Cell co-culture and cell migration assays

The co-culture model utilized the indirect contact co-culture approach. Mouse aortic endothelial cells (MAEC) and C3H10T 1/2 cell lines were obtained from the ATCC.

All cells are regularly tested for the presence of mycoplasma contamination. Pericytes (C3H10T 1/2 cell lines) and endothelial cells (MAEC), both exponentially growing, were specifically chosen for this study. Pericytes were introduced into a microporous chamber with a polycarbonate membrane featuring 0.4 μm pores, while endothelial cells were cultured in a 24-well plate. Following a 24-h incubation period in the cell culture incubator, DMEM medium supplemented with 10% fetal calf serum was introduced. The microporous chamber with the polycarbonate membrane was then positioned on top of the 24-well plate containing the endothelial cells, establishing the co-culture model.

Migration of ECs was examined by Scratch assays following the standard protocol. Cells were trypsinized, seeded into 6-well plates with 3 parallel wells for each group, and cultured to 90% confluence. Then, the cells were incubated in the medium with 2% FBS, and vertical scratches were made with a 100-μL tip. The cells were photographed at 0,6 and 12 h under an inverted microscope to measure cell migration distance.

### Histology and immunostaining

Kidney tissues were excised and rinsed in PBS and fixed in 10% formalin for 48h, embedded in paraffin. Five μm thick paraffin sections were stained with hematoxylin eosin and periodic acid-Schiff (PAS). The sections were scanned at ×200 magnification.

Immunohistochemistry and immunofluorescence were performed on formalin fixed and deparaffinized tissue sections. Immunohistochemical staining was carried out to evaluate pericyte and endothelial cells damage of the kidney tissues in the different groups. The sections were treated with the appropriate amount of endogenous peroxidase blocker and incubate for 10 min at room temperature (37 °C) to inactivate endogenous peroxidase activity. The slides were then blocked for 30 min in PBST containing 3% BSA (PBST-BSA) at 37 °C and incubated overnight at 4 °C with primary antibodies. Primary antibodies used in this study include PDGFR-β (ab313777, 1:100) and NG2 (55027-1-AP, 1:200). Slides were then washed 3 times in PBST (10 min at RT) and incubated for 2 h with the secondary antibody (1:200) in PBST at RT. We employed Fiji to conduct a semi-quantitative analysis of the tissue sections. This analysis of immunohistochemistry considers both the staining intensity and the proportion of positive cells. Positive reactions are defined as brown staining within the cells, and the counts are calculated as the ratio of positive cells to the total number of cells.

For immunofluorescence staining, the sections were incubated with primary antibodies overnight at 4 °C for CD31 (AF3628, 10 µg/mL) and PDGFR-β(ab313777,1:1000). The sections were incubated with fluorescently labeled secondary antibodies for 1 h at RT, and the sections were then counterstained with DAPI for 5-10 min at 37 °C to identify cell nuclei (Olympus).

### Transmission electron microscopy

Pericytes were exposed to TNF-α for 24 h. The cells were fixed with 2.5% glutaraldehyde at 4 °C for 2 h, followed by incubation with 2% OsO4 for 2–3 h at 4 °C. Then the pericytes were dehydrated in acetone gradients and embedded in Epon-Araldite resin. Ultrathin sections (100 nm) were then obtained using microtome Leica UC7 (Leica, Wetzlar, Germany). Sections were then stained with uranyl acetate and lead citrate. Transmission electron microscopy (TEM) was used to quantify the number of autophagosomes in pericytes.

### Pcr

PCR analyses were done in tissues that were stored in RNA later. Total RNA was isolated using an RNA Rapid Extraction Kit (RN001-50Rxns) according to the manufacturer’s instruction. Extracted RNAs were used for reverse-transcriptase PCR (RT-PCR) with High-Capacity cDNA Reverse Transcription Kit (Thermo fisher). Quantitive PCR (qPCR) was carried out using the LightCycler^®^ 480 SYBR Green I Master kit (Roche, Switzerland) in a LightCyler 480 (Roche, Switzerland).

The primer sequences were as follows: eNOS forward primer sequence (5′-3′),TCAGCCATCACAGTGTTCCC; eNOS reverse primer sequence (5′-3′), ATAGCCCGCATAGCGTATCAG; ICAM-A forward primer sequence (5′-3′), GTGATGCTCAGGTATCCATCCA; ICAM-A reverse primer sequence (5′-3′), CACAGTTCTCAAAGCACAGCG; GAPDH forward primer sequence (5′-3′), AATGGATTTGGACGCATTGGT; GAPDH reverse primer sequence (5′-3′), TTTGCACTGGTACGTGTTGAT.

### Western blot

Proteins were extracted from cells/tissue using RIPA lysis buffer. The supernatant was separated, and the protein concentration was measured and adjusted by the BCA method. Western blotting analysis of proteins was performed after samples had been resolved by SDS–polyacrylamide gel electrophoresis and transferred onto PVDF membranes. After blocking 5% milk, membranes were incubated with primary antibodies overnight. The primary antibodies used are: eNOS (CST, D9A5L, 1:1000), ICAM-1 (Proteintech, 10020-1-AP, 1:2000), GAPDH (Proteintech, 10494-1-AP, 1:5000), LC3B (CST, 2775S, 1:2000) and MCP-1 (Proteintech, 26161-1-AP, 1:1000). The membranes were washed in TBST and probed with the corresponding HRP-conjugated secondary antibody (anti-mouse or anti-rabbit). The gel imaging system was used to expose and scan strips to analyze the gray value of each band.

### Bioinformatics analysis

Gene Expression Omnibus (GEO) database series accession codes for data sets generated and used in this study are GSE 140988. The log2-transformed fold differences in expression and the p -values were calculated with GEO2R available at GEO website based on LIMMA package. The p -values were adjusted with Benjamini–Hochberg method (False Discovery Rate). For RNA expression in pericytes the adjusted *p < 0.05* was defined significant. KEGG pathway enrichment analysis was performed using the KEGG database.

### Statistical analysis

SPSS 22.0 and GraphPad Prism 9.0 were utilized for statistical analysis, while Image-Pro Plus 6.0 and Image J FiJi were employed for semi-quantitative analysis of chemical staining images. A significance level of *p < 0.05* was considered statistically significant. Data were presented as mean ± standard deviation (SD). The differences between the two datasets were analyzed using Pearson χ test or Student’s t test as appropriate. For multiple group comparisons, ANOVA one-way analysis of variance with Bonferroni correction was applied. Kaplan-Meier curve analysis and Cox regression analysis were utilized for single-factor and multi-factor analysis on factors influencing graft function.

## Results

### Analysis of prognosis and influencing factors of kidney transplantation in patients with ABMR

A total of 160 patients who underwent renal transplant were included in this cohort and categorized into four groups based on different histopathological results of renal biopsy: antibody-mediated rejection group (ABMR, *n* = 79), T cell-mediated rejection group (TCMR, *n* = 20), mixed rejection group (*n* = 25) combining ABMR and TCMR, and steady-state graft function group (STA, *n* = 36). Patients with other kidney diseases were excluded from the cohort. Table 1 displays the detailed clinical basic information of the patients. The Banff pathological rating table for pathological sections of kidney transplant recipients is presented in Table 2. Among all the scoring items, 10 out of 13 items showed statistically significant differences between groups (*p < 0.05*), and 5 items exhibited highly significant differences (i, t, ptc, g, mm, *p < 0.001*).

**Table 1.** Clinical characteristics of the analysis groups.

		ABMR	TCMR	Mixed	STA	*P* value
Patients, *n*		79	20	25	36	
Sex, *n*	Male	64	17	17	27	0.033
	Female	15	3	8	9	
Age		46.15 ± 7.44	42.2 ± 3.72	38.99 ± 6.99	49.42 ± 6.81	0.085
Donor type, *n*	Deceased	73	17	21	36	0.015
	Living, related	6	3	4	0	
DGF	Yes	24	6	8	3	0.273
	No	55	14	17	33	
Post-transplantation time, year		8.29 ± 1.13	7.2 ± 1.06	8.25 ± 1.54	6.51 ± 0.42	<0.001
HLA match		0.18 ± 0.16	0.22 ± 0.16	0.21 ± 0.2	0.18 ± 0.14	0.924
DSA	Positive	31	7	11	6	0.06
	Negative	48	13	14	30	
PRA	Positive	30	7	8	17	<0.001
	Negative	49	13	17	19	
Immunotherapy regimen	Triple-drug immunosuppression	51	13	15	27	0.046
	Triple-drug immunosuppression + Sirolimus	13	3	6	2	
	Triple-drug immunosuppression + Iguratimod	5	4	3	4	
	Other	10	0	1	3	

ABMR: antibody-mediated rejection; TCMR: T-cell-mediated rejection; DGF: delayed graft function; HLA: human leucocyte antigen; DSA: donor-specific antibody; PRA: panel reactive antibody; triple-drug immunosuppression: cyclosporine, azathioprine, and prednisolone.

**Table 2.** Histopathological diagnosis of the renal biopsies according to the BANFF classification.

	ABMR	TCMR	Mixed	STA	*p* value
i	0.83 ± 0.99	1 ± 0.71	2.09 ± 1.22	0.08 ± 0.28	<0.001
t	0.27 ± 0.45	0.26 ± 0.45	0.82 ± 0.41	0.13 ± 0.34	<0.001
v	0.13 ± 0.57	0.2 ± 0.45	0.36 ± 0.51	0	0.006
ptc	1 ± 0.74	0.8 ± 0.45	1.09 ± 0.3	0.21 ± 0.42	<0.001
g	0.53 ± 0.51	0.8 ± 0.45	1.18 ± 0.41	0.13 ± 0.34	<0.001
C4d	0.4 ± 0.68	0.2 ± 0.45	0 ± 0	0.13 ± 0.34	0.085
cg	0.07 ± 0.25	0	0.27 ± 0.47	0	0.01
ci	0.17 ± 0.38	0	0.18 ± 0.41	0	0.013
ct	0.13 ± 0.35	0	0.18 ± 0.41	0.08 ± 0.28	0.086
cv	0.03 ± 0.18	0	0.27 ± 0.47	0	0.002
ah	0.13 ± 0.35	0.4 ± 0.55	0.36 ± 0.51	0	0.002
mm	0.03 ± 0.18	0	0.82 ± 1.4	0	<0.001
ti	0.05 ± 0.36	0.05 0.38	0.27 ± 0.91	0	0.089

The abbreviations: i for interstitial inflammation of nonfibrotic areas of the cortex, t for tubulotubulitis of the cortical area, v for endoarteritis (intimal arteritis), ptc for peritubular capillaritis, g for glomerulitis, C4d for positive immunofluorescence staining or immunohistochemical staining of C4d peritubular capillaries, cg for chronic glomerulopathy (transplant glomerulopathy), ci for cortical interstitial fibrosis, ct for cortical tubular atrophy, cv for intimal fibrosis (thickening of the fibrous intima), ah for arteriolar hyalinopathy, mm for glomerular capillary mesangial matrix proliferation, and ti for global cortical interstitial inflammation. Data were expressed as mean ± SD.

**Figure 1.**
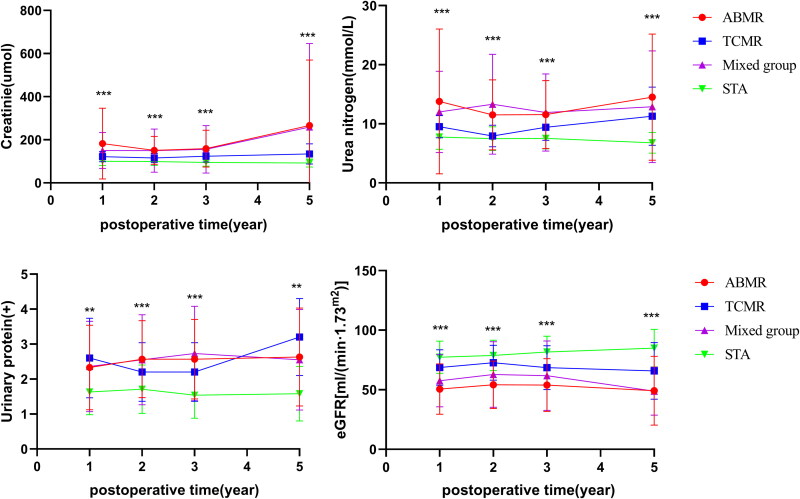
The renal function of patients in each group was assessed at 1-, 2-, 3-, and 5-years post-transplantation. The levels of creatinine, urea nitrogen, urinary protein and eGFR exhibited changes in all groups at 1-, 2-, 3-, and 5-years post transplantation. (**: *p < 0.01*, ***: *p < 0.01*, ****p < 0.0001)

Post-transplantation recipients’ serum creatinine (sCr) (umol/L), urea nitrogen (mmol/L), urinary protein and the estimated glomerular filtration rate (eGFR) of patients in each group are present in Figure 1 and Table S1.

The results indicate significant differences in Scr and BUN levels among the four groups (*p < 0.001*), as well as in urinary protein levels (*p < 0.05*) one-year post-surgery, with variations observed at two, three, and five years. Among the laboratory data, the ABMR group exhibited the highest serum creatinine levels and the lowest eGFR compared to the other groups. Notably, eGFR values differed significantly among the groups (*p < 0.001*), with a decrease observed in all groups relative to the control group, particularly in the ABMR group, suggesting more pronounced renal function impairment in cases of antibody-mediated rejection (Figure 1). Table 2 presents the multiple comparison results of eGFR, revealing statistically significant differences between the ABMR group and the control group (*p < 0.001*), as well as between the mixed group and the control group (*p < 0.05*). The disparity in eGFR between the ABMR group and the control group increased over time post-transplantation. While variations in eGFR were noted among other groups, these differences did not reach statistical significance (Table S1).

**Figure 2.**
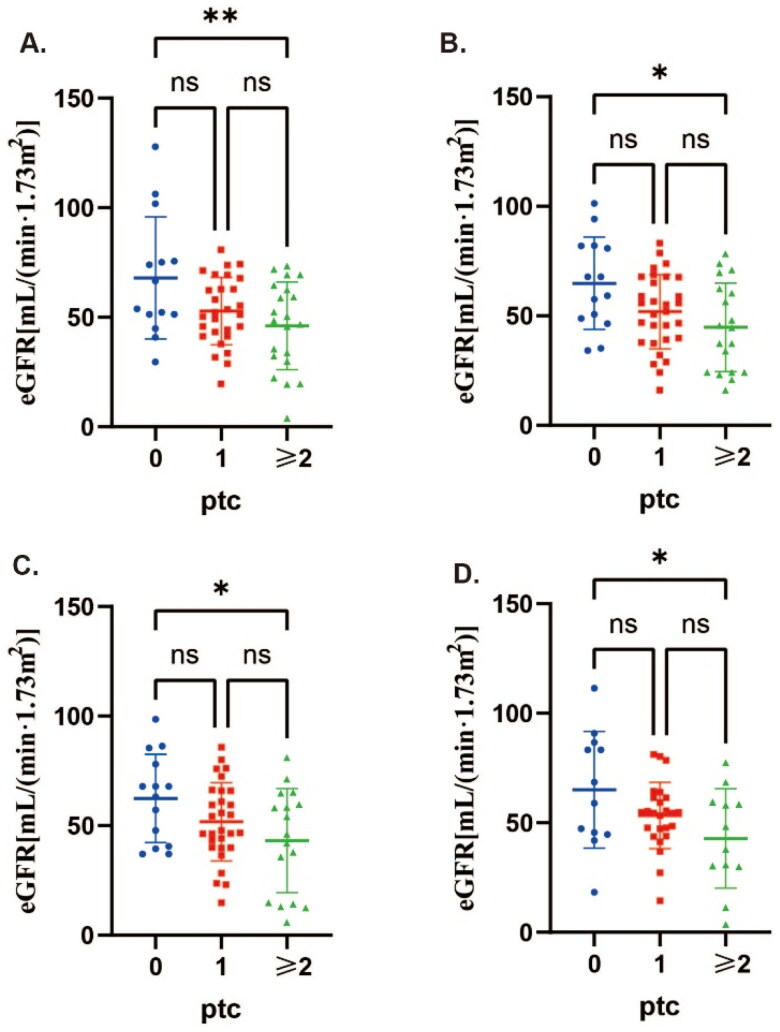
The ptc score showed a significant correlation with renal function post-transplantation. A. The correlation between ptc score and renal function in pathological specimens of ABMR patients within the initial year post renal transplantation. B. The correlation between ptc score and renal function in pathological specimens of ABMR patients within the second year post renal transplantation. C. The correlation between ptc score and renal function in pathological specimens of ABMR patients within the third year post renal transplantation. D. The correlation between ptc score and renal function in pathological specimens of ABMR patients within the fifth year post renal transplantation. The difference was statistically significant. (*: *p < 0.05*; **: *p < 0.01*)

The KM curve univariate analysis results (Table S2) indicated that post-transplantation time, DSA, immunotherapy regimen, pre-renal puncture renal function, v, ptc, and ct were significant factors influencing the prognosis of kidney transplantation in ABMR patients (*p < 0.05*). When comparing the renal function levels of ABMR patients across different ptc score groups (ptc = 0, ptc = 1, ptc ≥ 2) post-transplantation (Figure 2, Table S3-S4) at the 1st, 2nd, 3rd, and 5th years after surgery, significant differences were observed. The renal function was found to be better in the ptc = 0 group and worse in the ptc ≥ 2 group. Multiple comparison results (Table S5) further confirmed the statistical differences between the ptc = 0 and ptc ≥ 2 groups. Additionally, the results of the Cox multivariate regression analysis (Table S4) highlighted that the time post-transplantation and pre-renal puncture renal function level were the only influencing factors (*p < 0.05*).

**Table 3.** Clinical information for patients in three groups.

		Total	ABMR	TCMR	STA	*P* value
Age,year		40.89 ± 12.81	37.33 ± 14.05	39.33 ± 7.37	46 ± 18.52	0.743
Sex	Male	6	2	1	3	0.679
	Female	3	1	2	0	
Donor kidney source	deceased-donor kidney	6	3	1	2	0.679
	related-donor kidney	3	0	2	1	
DSA	Positive	4	2	1	1	1
	Negative	5	1	2	2	
HLA match	<50%	6	3	1	2	0.679
	≥50%	3	0	2	1	
Transplatotion, year		4.78 ± 2.05	6.67 ± 0.58	4 ± 2.65	3.67 ± 1.16	0.138
DGF	Yes	0	0	0	0	–
	No	9	3	3	3	
Immunotherapy regimen	Triple-drug immunosuppression	4	2	1	1	1
	Triple-drug immunosuppression + Sirolimus	2	0	1	1	
	Triple-drug immunosuppression + Iguratimod	1	1	0	0	
	Other	2	0	1	1	

**Table 4.** Estimated glomerular filtration rate(eGFR)(ml/min)in patients from three groups.

	Total	ABMR	TCMR	STA	*P* value
eGFR after aspiration biopsy	63.44 ± 30.67	28.88 ± 17.85	72.49 ± 19.64	88.97 ± 12.88	0.012
1-year eGFR after kidney transplantation	79.14 ± 20.01	66.24 ± 27.35	88.34 ± 20.69	82.84 ± 5.35	0.425
2-year eGFR after kidney transplantation	74.59 ± 23.55	69.63 ± 40.54	77.66 ± 15.75	77.49 ± 10.75	0.926
3-year eGFR after kidney transplantation	73.66 ± 25.96	66.72 ± 40.45	86.54 ± 26.78	72.03 ± 11.05	0.763
5-year eGFR after kidney transplantation	51.8 ± 33.23	25.44 ± 19.9	71.05 ± 16.08	92.37 ± 11.53	0.083

**Figure 3.**
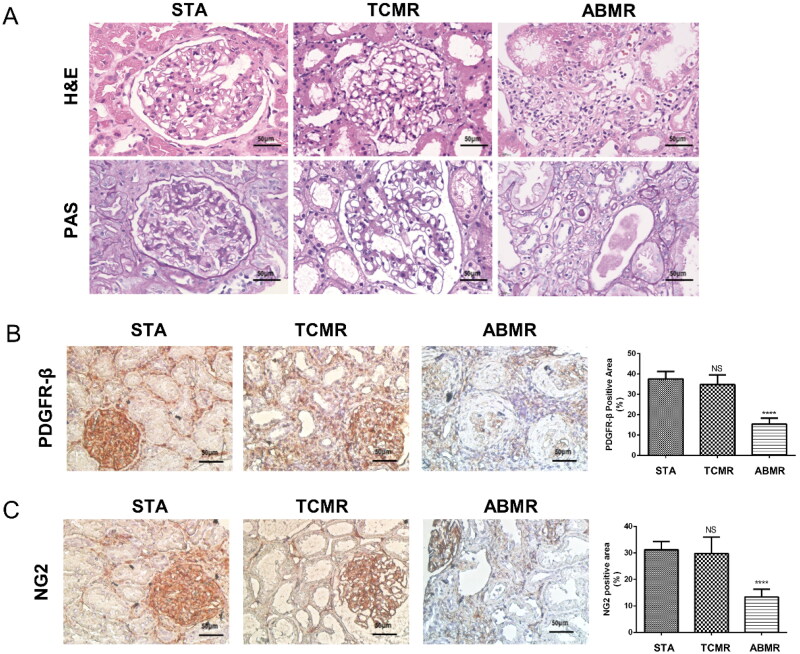
Significant damage to peritubular capillaries (PTC) and dysfunction of microvascular pericytes were noted in transplanted kidney tissue with ABMR. A. HE and PAS staining in renal puncture pathological specimens from kidney transplant patients; B. Immunohistochemical staining of PDGFR-β and semi-quantitative analysis results of PDGFR-β immunohistochemical staining positive areas in renal puncture pathological specimens from kidney transplant patients (NS indicates no statistically significant difference when compared to the STA group; ****: *p < 0.0001* versus STA.); C. Immunohistochemical staining of NG-2 and semi-quantitative analysis results of NG-2 immunohistochemical staining positive areas in renal puncture pathological specimens from kidney transplant patients. (NS indicates no statistically significant difference when compared to the STA group; ****: *p < 0.0001* versus STA.)

Three renal biopsy specimens were obtained from transplant patients in the ABMR group, TCMR group, and STA group. Immunohistochemical staining was conducted to assess the status of PDGFR-β and NG2-positive pericytes in each group (Figure 3(B,C)). The results revealed higher expression of PDGFR-β and NG2 in the STA group compared to the ABMR and TCMR groups, indicating a significant impairment in pericyte function in the context of ABMR.

### Microvascular endothelial cells damage and microvascular pericytes dysfunction were observed in allograft with ABMR

Renal biopsy specimens were collected from 3 transplant patients in each of the ABMR group, TCMR group, and STA group for immunohistochemical staining. Clinical data and Banff score results for these patients are presented in Tables 3,4, S6.

Hematoxylin and Eosin staining and PAS staining results of pathological specimens from patients in the STA group revealed slight proliferation of mesangial cells and a slight increase in mesangial matrix in the transplanted kidney (Figure 3A). Capillary openings were normal, with occasional mononuclear cell infiltration in the loops, but no microthrombi were observed. Renal tubulointerstitial lesions were mild, with some individual tubules showing atrophy, thickening of the basement membrane, and a small amount of mononuclear cell infiltration in the interstitium. Arteriolar smooth muscle cells exhibited vacuolar degeneration and occasional hyaline degeneration. In the ABMR group, there was infiltration of mononuclear cells and eosinophils in multiple peritubular capillaries, mononuclear cell infiltration and tubulitis in the interstitium of tubules, irregular thickening of glomerular capillary basement membrane, and swelling and proliferation of endothelial cells.

**Figure 4.**
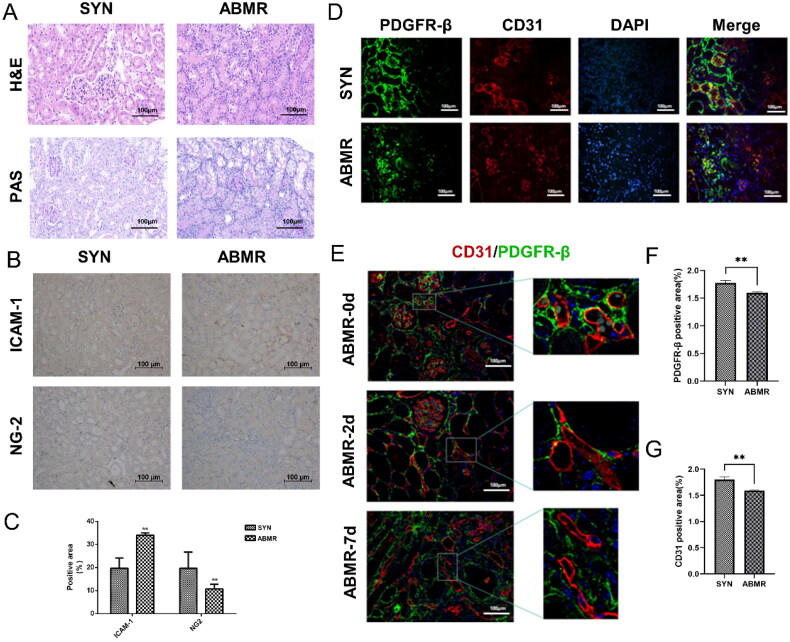
Significant damage to peritubular capillaries (PTC) and dysfunction of microvascular pericytes were noted in kidney specimens of mouse kidney transplant model. A. HE and PAS staining in kidney specimens of mouse kidney transplant model; B. Immunohistochemical staining of NG2 and ICAM-1 markers in kidney specimens of mouse kidney transplant model; C: Semi-quantitative analysis of immunohistochemically positive areas of mouse transplanted kidney tissue sections; D. Immunofluorescence staining of mouse transplanted kidney tissue sections; E. Continuous fluorescent staining chart of kidney specimens of ABMR model of mouse kidney transplantation. F-G: Semi-quantitative analysis of immunofluorescent staining of PDGFR-β+ and CD31+ in mouse transplanted kidney tissue sections. (**: *p < 0.01*).

A mouse kidney transplant model was established for the study. In the allograft tissues, the SYN group exhibited signs of ischemia-reperfusion injury and interstitial edema. The transplanted glomeruli and renal tubule structures appeared normal without significant mononuclear cell infiltration. Additionally, no notable expansion was observed in the renal tubules and collecting system. In contrast, HE staining of the transplanted kidneys from the ABMR group revealed acute damage to the glomeruli and renal tubules, including loss of the brush border in the tubules and lumen dilation. Peritubular capillaries were markedly dilated and congested, with infiltration of numerous neutrophils and mononuclear cells. Segmented nuclei were visible in the interstitium, indicating lymphocyte infiltration. Lymphocyte infiltration was scattered around arteries, with some blood vessels displaying transmural arteritis (Figure 4(A)). PAS staining further confirmed neutrophil and mononuclear cell infiltration in the peritubular capillaries between the renal tubular basement membranes. Mononuclear cell infiltration was also evident in certain renal tubules and glomeruli, suggesting tubulitis and glomerulitis (Figure 4(A)). Immunohistochemistry results indicated that the SYN group exhibited higher levels of positive expression of NG2 and lower levels of positive expression of ICAM-1 (Figure 4(B)). These findings suggested that the functions of pericytes and endothelial cells in the ABMR transplanted kidney were notably imparied.

To investigate changes in the distribution of juxtaglomerular vascular endothelium and pericytes in ABMR transplanted kidneys, we utilized FITC-labeled PDGFR-β and Cy3-labeled CD31 for detecting pericytes and endothelial cells in kidney section specimens of a kidney transplant mouse model. Double immunofluorescence staining was conducted, revealing that in the presence of ABMR, the vascular endothelium’s morphology is compromised, leading to disruption in continuity and detachment of pericytes from the endothelium. In the SYN group’s transplanted kidneys, the vascular endothelium and pericytes exhibited close association, whereas in the ABMR group’s transplanted kidney specimens, reduced expressions of the vascular endothelial marker CD31 and the pericyte marker PDGFR-β were observed, with some pericytes detaching from endothelial cells and migrating to the interstitial region, resulting in green fluorescence in the renal interstitium (Figure 4(C-G)). These differences were found to be statistically significant.

### Microvascular pericyte dysfunction results in damage to vascular endothelial cells

**Figure 5.**
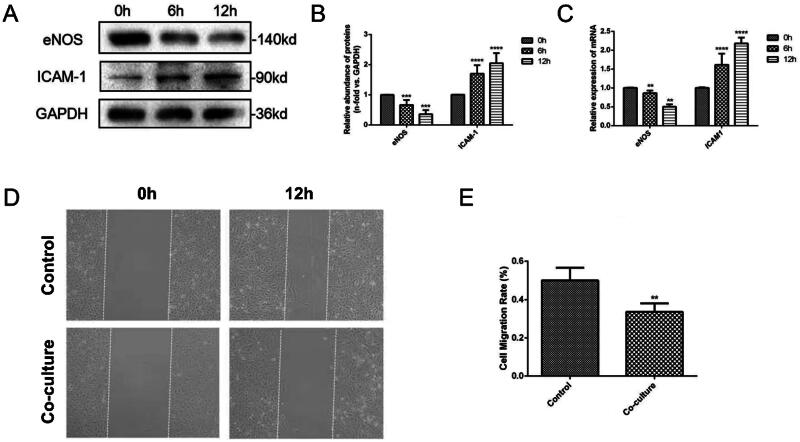
Microvascular pericyte dysfunction can result in endothelial injury. A cell model of microvascular pericyte dysfunction was created and co-cultured with vascular endothelial cells. A. The expression of eNOS and ICAM-1 in endothelial cells was detected using Western blot technology post co-culture. B. Semi-quantitative analysis of Western blot results was conducted. C. The PCR method was employed to discern disparities in eNOS and ICAM-1 expression in endothelial cells pre- and post-co-culture. D. Statistical analysis chart of cell migration rates in the control group and co-culture group was generated. E. Cell scratch test chart of the control group and co-culture group was also examined. (*: *p < 0.05*, **: *p < 0.01*, ***: *p < 0.001*, ****p < 0.0001).

A co-culture model of vascular endothelial cells and pericyte dysfunction cells was utilized. Endothelial cells were collected at 0, 6, and 12 h for Western blot analysis to detect changes in eNOS (endothelial nitric oxide synthase) and ICAM-1 (intercellular adhesion molecule 1) expression (Figure 5(A)). Semi-quantitative analysis revealed that with longer co-culture times, there was a decrease in eNOS expression (*p* < 0.001) and an increase in ICAM-1 expression (*p* < 0.0001) in endothelial cells (Figure 5(B)). The mRNA expression of eNOS and ICAM-1 in endothelial cells was also measured using the PCR method, showing similar expression patterns as observed in the Western blot analysis (Figure 5(C)). Additionally, the wound healing assay demonstrated that after 12 h of co-culture, the migration rate of endothelial cells was lower in the co-culture group compared to the control group (*p* < 0.01) (Figure 5(D)). Collectively, these findings indicate that pericyte dysfunction can disrupt the normal physiological functions of vascular endothelial cells. The above experiments were repeated three times with similar results.

### Autophagy plays a crucial role in pericyte dysfunction

**Figure 6.**
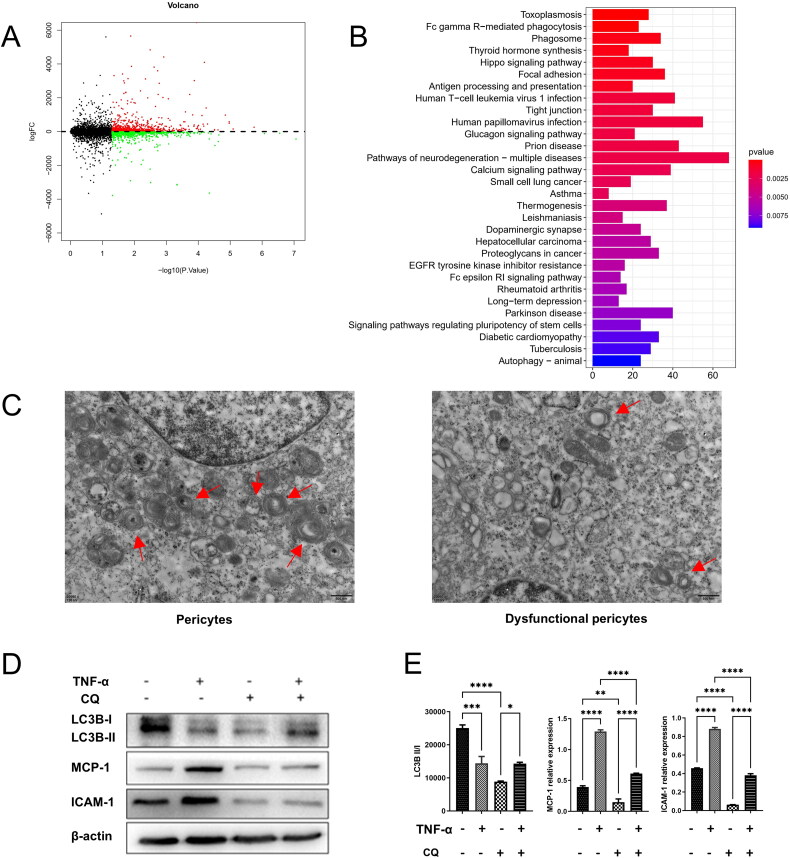
Autophagy plays a crucial role in pericyte dysfunction. A. Volcano plot of differentially expressed genes in pericellular dysfunction. Red dots indicate the upregulated proteins; Green dots show the downregulated proteins. DEPs with at least 2.0-FC and p-value less than 0.01 are shown in red or green color; B. The KEGG analysis of differentially expressed genes in pericellular dysfunction; C. Autophagosomes in pericytes (left) and dysfunctional pericytes (right) are detected using transmission electron microscopy (TEM) (Red arrow: autophagosomes); D. The expression of LC3B, ICAM-1, and MCP-1 in peripheral cells of each group was assessed using western blot analysis; E. Semi-quantitative analysis of Western blot results was conducted. (*: *p < 0.05*, **: *p < 0.01*, ***: *p < 0.001*, ****p < 0.0001).

To investigate the underlying molecular mechanisms, we utilized the GEO database to analyze pericytes from the kidney pre and post-acute kidney injury. Differential gene expression analysis was performed on activated and dysfunctional pericytes (Figure 6(A)), revealing significant changes in the autophagy pathway based on KEGG analysis (Figure 6(B)). Subsequent transmission electron microscopy examination of autophagosomes in the pericyte dysfunction model demonstrated structural alterations such as swollen and blurred mitochondria, vacuolated cristae, and reduced autophagosome numbers (Figure 6(C)). Additionally, Western blot analysis revealed increased expression of ICAM-1 and MCP-1, decreased levels of LC3B, and reduced autophagy in tumor necrosis factor-alpha (TNF-α)-induced pericyte dysfunction. Conversely, inhibiting autophagy post-QC treatment led to decreased ICAM-1 and MCP-1 expression, reduced pericyte damage, and highlighted the significance of diminished autophagy in pericyte dysfunction (Figure 6(D,E)).

## Discussion

Kidney transplantation is a highly effective treatment for end-stage renal disease, greatly enhancing patients’ quality of life and prognosis [22]. Advances in immunosuppressive agents have led to a decrease in early postoperative T cell-mediated rejection (TCMR) rates, improving short-term survival rates to over 95% [23]. However, long-term survival rates still require enhancement. Recent studies have highlighted the impact of antibody-mediated rejection (ABMR) on long-term graft function in kidney transplant recipients, emerging as a primary cause of graft loss [24]. Moreover, evidence suggests greater economic burden and increased HRQOL impairment with ABMR-related kidney transplant rejection relative to non-ABMR, although small sample sizes and missing definitions for ABMR make meaningful comparisons between studies challenging [25]. The occurrence is not a matter of chance. Our clinical data analysis revealed that patients in the ABMR group exhibited poorer post-transplant renal function compared to those in the TCMR, mixed, and control groups. Furthermore, transplant recipients experiencing ABMR had significantly reduced long-term prognosis for kidney function.

The primary mechanism of ABMR following renal transplantation involves the activation and differentiation of B lymphocytes into plasma cells, leading to the production of donor-specific antibodies (DSA) that cause inflammatory damage to the graft endothelium through complement-dependent or complement-independent pathways [26–27]. According to the Banff pathological diagnostic criteria, ABMR diagnosis focuses on three main aspects: microvascular inflammation in the transplanted kidney, C4d deposition in peritubular capillaries, and detection of DSA in the circulation [28–29]. Previous research has demonstrated a significant negative correlation between the degree of endothelial cell damage in the transplanted kidney during ABMR and the long-term prognosis of the transplant [30–31]. Our study examined clinical data from 160 transplant patients and found a correlation between the peritubular capillaritis (PTC) score in the ABMR group and post-transplant renal function. Higher PTC scores were associated with poorer renal function postoperatively. This highlights the critical role of vascular endothelial cell damage in ABMR, its impact on microvascular disease in the transplanted kidney, and its significance for long-term transplant prognosis. Once ABMR develops, it is irreversible. Therefore, ongoing research is focused on understanding the mechanisms of renal vascular endothelial damage in ABMR post-kidney transplantation and identifying strategies to mitigate this damage and delay renal fibrosis progression to enhance the long-term outcomes of kidney transplants [32].

Perinephric cells, originating from mesenchyme, express platelet-derived growth factor receptor-β (PDGFRβ) and collagen. Pericytes attach to capillaries in the common basement membrane, forming connections with endothelial cells to support the microvasculature, regulate blood flow, and produce erythropoietin [33]. Involved in tissue repair, pericytes are vital for maintaining the normal operation of the microvasculature system and are closely linked to endothelial cells. This interaction enables pericytes to influence endothelial cells by releasing various factors such as Notch3, VEGF, angiopoietin 1, and PDGF-BB, leading to dynamic growth, migration, and phenotypic changes [34–35]. Dysfunction of pericytes can impact the physiological functions of endothelial cells. Research by Kramann et al. has demonstrated that pericyte dysfunction can result in endothelial damage and decreased peritubular capillary density [36]. Studies have also shown that detachment of pericytes from peritubular endothelial cells can cause endothelial cell injury, damage to the capillary network, and contribute to the progression of renal fibrosis [37].

Pericytes are recognized as precursors of myofibroblasts in the pathogenesis of tissue fibrosis [38]. Research indicates that renal ischemia-reperfusion injury can induce pericyte dysfunction, leading to their transition into myofibroblasts [17]. Endothelial cell dysfunction, loss of peritubular capillaries (PTCs), activation of myofibroblasts, and impaired repair of renal tubular epithelial cells are critical mechanisms underlying renal fibrosis. Among these, PTC damage and reduced density worsen renal fibrosis progression, with ongoing fibrosis further compromising PTCs [39]. Renal pericytes serve as a crucial link between PTC damage and interstitial fibrosis. Nevertheless, the precise involvement and function of pericytes in PTC damage attributed to antibody-mediated rejection (ABMR) and the subsequent fibrotic processes remain inadequately understood. Therefore, our study aims to investigate the specific role of pericytes in ABMR-induced PTC damage and its potential impact on fibrosis.

Immunohistochemical staining results of renal pathological specimens from patients and mice confirmed that pericytes were reduced in the ABMR group compared with the STA group. Additionally, immunohistochemical staining results of ICAM-1 in mouse transplanted kidney tissue sections indicated higher expression of ICAM-1 in the ABMR group than in the STA group. Immunofluorescence staining further revealed a decrease in the number of pericytes in the ABMR group, with some pericytes detaching from the endothelium. ICAM-1 in endothelial cells enhances the adhesion of various cells through cell-cell interactions, facilitating cell migration across the endothelium and contributing to inflammatory and immune responses. Normally, ICAM-1 expression is low on resting vascular endothelial cells but increases during inflammatory and immune responses, aiding in the clearance of foreign antigens and tumor cells [40–41]. These findings suggest that in ABMR, pericytes detach from the vascular endothelium, migrate, and undergo transformation, losing their normal physiological functions and cell phenotypes. Furthermore, endothelial cell dysfunction in ABMR transplanted kidney tissue impairs the peritubular capillary network, ultimately leading to a decline in renal function [42].

A co-culture model of pericytes and vascular endothelial cells was developed *in vitro* to study their interaction. The results revealed significant changes in the expression levels of eNOS and ICAM-1 in endothelial cells when co-cultured with dysfunctional pericytes. Nitric oxide (NO) plays a crucial role in regulating vasodilation and inhibiting various processes in the vascular endothelium. When eNOS expression is reduced or impaired, the bioavailability of NO decreases, leading to an imbalance in vascular homeostasis [43]. The findings after co-culture showed a decrease in eNOS expression and migration ability of endothelial cells, along with an increase in ICAM-1 expression, suggesting that dysfunctional pericytes can induce damage to endothelial cells, potentially contributing to the development of ABMR and kidney transplant failure. Notch3 activation in pericytes of ABMR transplanted kidneys has been associated with negative transplant outcomes, highlighting the importance of addressing pericyte dysfunction as a potential treatment strategy to prevent the progression of acute kidney injury to chronic kidney disease post-ABMR [44]. Further research on drugs targeting pericytes could offer promising avenues for improving the treatment of ABMR following kidney transplantation [45].

To investigate the molecular mechanisms underlying pericyte dysfunction, we utilized the GEO database for our study. Specifically, we conducted a genome-wide analysis (GSE140988) on pericytes extracted from the kidney both before and after acute kidney injury [46]. Our analysis focused on differential gene expression between activated and dysfunctional pericytes. Subsequent KEGG analysis revealed significant alterations in the autophagy pathway. Autophagy, a crucial cellular process, relies on the lysosomal pathway to break down damaged organelles and denatured proteins [47]. This mechanism serves as a protective response in cells, helping them adapt to adverse conditions and maintain long-term cellular homeostasis. Autophagy also plays a key role in modulating inflammation and immune responses in the body [48]. Previous research has highlighted the protective effects of autophagy in conditions like acute kidney injury [34]. Notably, studies have demonstrated that chloroquine, a lysosome inhibitor, can disrupt autophagy flux by preventing the fusion of autophagosomes with lysosomes [49]. Inhibition of autophagy with chloroquine has been linked to exacerbation of cisplatin-induced acute kidney injury. This blockade in lysosomal degradation of autophagosomes leads to increased cell death, tissue damage, and ultimately, deterioration of renal function [50].

The impact of autophagy machinery on the dynamic activity of pericytes within vascular structures has been a topic of debate. Using transmission electron microscopy, autophagosomes were detected preliminarily. Results indicated that in a pericyte dysfunction model, pericyte ultrastructure was compromised, with swollen and blurred mitochondria, vacuolated or disappearing cristae, and blurred Z lines. The number of autophagosomes significantly decreased. Western blot results in a TNF-α-induced pericyte dysfunction model showed reduced autophagy levels, with pericyte damage reduced after inhibiting autophagy with QC. These findings suggest that autophagy defects play a crucial role in pericyte dysfunction. Prior research has linked autophagy to pericyte function in various blood vessel types, with autophagy abnormalities contributing to pericyte dysfunction in different pathological conditions [51]. Studies on diabetes have shown that retinal pericyte loss can impair blood-retinal barrier function and increase vascular leakage, where autophagy may regulate retinal vascular cell stability through its impact on pericytes [52]. Additionally, under inflammatory conditions, autophagy can influence the differentiation potential of pericytes. However, the precise role of autophagy in guiding progenitor cells toward functional pericytes remains unclear and necessitates further investigation. Hassanpour and colleagues further validated the involvement of the autophagy mechanism in the differentiation of CD146 pericyte progenitor cells into mature pericytes and ECs [53]. It is theorized that the initiation of an autophagy response may be triggered by the production of pro-inflammatory cytokines.

Impaired autophagy machinery can result in pericyte dysfunction in inflammatory conditions. Yuan Zhang et al. found that knocking out sigma-1 receptor (σ-1 R) can cause pericyte loss by increasing autophagy and apoptosis, with σ-1 R playing a role in the interplay between apoptosis and autophagy, guiding pericytes toward survival [54]. Activation of adaptive autophagy can aid in the recovery of injured pericytes’ connections with ECs, but excessive autophagy activation may lead to pericyte apoptosis. Overall, autophagy can have a protective effect on pericytes in a time- and intensity-dependent manner. What’s more, the mechanisms by which adaptive autophagy regulates pericyte function, enhances resistance to immune disorders and inflammatory storms post-kidney transplantation, and restores cellular homeostasis remain unexplored. This presents a challenge for future research. Furthermore, the crucial role of various forms of autophagy in the dynamic activity of peritubular cells and endothelial cells requires further exploration. Our study aims to investigate the interaction between peritubular cells and endothelial cells through *in vitro* cell culture, knockout mouse models of autophagy-related genes, drug intervention experiments, and related genomic technologies. This will enable us to deeply study the molecular mechanisms of autophagy regulating the interaction between peritubular cells and endothelial cells, as well as its detailed effects on their transformation. Additionally, we will focus on detecting peritubular cell function disorders in clinical practice and explore how targeted regulation of peritubular cell adaptive autophagy can be applied to treat antibody-mediated rejection (ABMR) after kidney transplantation.

However, it is important to acknowledge certain limitations in our study. While the sample size (160 patients) and research period (2004–2020) are relatively sufficient for a retrospective study, further prospective studies are needed to better explore the effects of peritubular cell function disorders on ABMR after kidney transplantation. Furthermore, considering the differences in physiology and immune responses between humans and mice, there are limitations to using an ABMR mouse model. It remains unknown whether database bioinformatics analysis of mouse peritubular cells can yield similar results in humans; therefore, translating these findings into clinical applications—particularly regarding the regulation of peritubular cell autophagy for treating ABMR after kidney transplantation—still requires extensive research efforts.

## Conclusion

In this study, an analysis of clinical data from kidney transplant patients at our center revealed a significant reduction in the long-term prognosis of allograft in recipients with ABMR. The occurrence of ABMR was closely associated with vascular injury, with noticeable vascular injury and dysfunction of pericyte cells observed in the transplanted kidney tissue of ABMR patients, the dysfunction of which may serve as a key mechanism for long-term dysfunction induced by ABMR. The study suggests that autophagy disorder could be a significant factor contributing to pericyte dysfunction.

## Supplementary Material

Supplemental Material

## Data Availability

The datasets during and/or analyzed during the current study are available from the corresponding author on reasonable request.
