# Supplementary material for: Role of microvascular pericyte dysfunction in antibody-mediated rejection following kidney transplantation
Source: Ren Fail. 2025 Feb 5;47(1):2458749. doi: 10.1080/0886022X.2025.2458749 (PMC11803764; doi:10.1080/0886022X.2025.2458749)
Supplement: Supplemental Material [file IRNF_A_2458749_SM7914.docx]

**Supplementary tables**

**Table S1.** Comparison of eGFR in pairs between groups (ml/min)

|  |  | Mean difference | Standard deviation, SD | *P* value | 95% confidence interval, 95% CI | |
| --- | --- | --- | --- | --- | --- | --- |
|  |  |  |  |  | The lower limit | The upper limit |
| 1-year eGFR after kidney transplantation | ABMR vs TCMR | -10.5 | 5.44 | 0.333 | -25.04 | 4.05 |
|  | ABMR vs Mixed | -2.63 | 4.96 | 1 | -15.89 | 10.64 |
|  | ABMR vs STA | -23.32 | 4.28 | **＜0.001** | -34.77 | -11.86 |
|  | TCMR vsMixed | 7.87 | 6.53 | 1 | -9.58 | 25.33 |
|  | TCMR vsSTA | -12.82 | 6.03 | 0.211 | -28.94 | 3.3 |
|  | MixedvsSTA | -20.69 | 5.6 | **0.002** | -35.67 | -5.71 |
| 2-year eGFR after kidney transplantation | ABMR vs TCMR | -14.3 | 5.6 | 0.07 | -29.27 | 0.67 |
|  | ABMR vsMixed | -5.37 | 5.11 | 1 | -19.03 | 8.29 |
|  | ABMR vsSTA | -25.72 | 4.41 | **＜0.001** | -37.52 | -13.93 |
|  | TCMR vsMixed | 8.93 | 6.71 | 1 | -9.01 | 26.87 |
|  | TCMR vsSTA | -11.43 | 6.2 | 0.403 | -27.99 | 5.14 |
|  | MixedvsSTA | -20.35 | 5.76 | **0.003** | -35.75 | -4.96 |
| 3-year eGFR after kidney transplantation | ABMR vs TCMR | -11.87 | 6.22 | 0.349 | -28.5 | 4.76 |
|  | ABMR vsMixed | -3.67 | 5.65 | 1 | -18.79 | 11.45 |
|  | ABMR vsSTA | -26.98 | 4.87 | **＜0.001** | -40 | -13.97 |
|  | TCMR vsMixed | 8.2 | 7.41 | 1 | -11.64 | 28.03 |
|  | TCMR vsSTA | -15.11 | 6.83 | 0.171 | -33.39 | 3.17 |
|  | MixedvsSTA | -23.31 | 6.32 | **0.002** | -40.23 | -6.39 |
| 5-year eGFR after kidney transplantation | ABMR vs TCMR | -12.72 | 7.2 | 0.479 | -32.04 | 6.6 |
|  | ABMR vsMixed | 2.64 | 5.96 | 1 | -13.35 | 18.63 |
|  | ABMR vsSTA | -32.61 | 5.51 | **＜0.001** | -47.41 | -17.82 |
|  | TCMR vsMixed | 15.36 | 8.33 | 0.406 | -6.99 | 37.71 |
|  | TCMR vsSTA | -19.89 | 8.02 | 0.087 | -41.4 | 1.62 |
|  | MixedvsSTA | -35.25 | 6.92 | **＜0.001** | -53.83 | -16.68 |

**Table S2.** Univariate analysis affecting the prognosis of kidney transplantation (K-M curve method)

| Influence factor | χ^2^ | Degree of freedom, df | *P* value |
| --- | --- | --- | --- |
| Sex | 0.206 | 1 | 0.650 |
| Age | 0.896 | 2 | 0.639 |
| Donor type | 0.729 | 1 | 0.393 |
| DGF | 1.07 | 1 | 0.744 |
| Transplantation time | 12.950 | 2 | **0.002** |
| HLA match | 0.331 | 1 | 0.565 |
| DSA | 4.604 | 1 | **0.032** |
| PRA after transplatation | 0.000 | 1 | 0.996 |
| Immunotherapy regimen | 19.066 | 3 | **＜0.001** |
| Renal function after aspiration biopsy | 11.335 | 3 | 0.010 |
| i | 0.663 | 3 | 0.882 |
| t | 0.072 | 1 | 0.788 |
| v | 7.514 | 2 | 0.023 |
| ptc | 7.884 | 3 | 0.048 |
| g | 0.475 | 2 | 0.789 |
| C4d | 1.220 | 3 | 0.748 |
| cg | 0.020 | 1 | 0.887 |
| ci | 2.686 | 1 | 0.101 |
| ct | 12.759 | 2 | **0.002** |
| cv | 0.349 | 1 | 0.530 |
| ah | 0.013 | 1 | 0.910 |
| mm | 3.670 | 2 | 0.160 |
| ti | 0.359 | 1 | 0.549 |

**Table S3**. Changes in estimated glomerular filtration rate (eGFR) (ml/min) among ABMR patients within each ptc score group.

|  | 1-year after transplantation | | 2-year after transplantation | | 3-year after transplantation | | 5-year after transplantation | |
| --- | --- | --- | --- | --- | --- | --- | --- | --- |
|  | N | eGFR | N | eGFR | N | eGFR | N | eGFR |
| ptc=0 | 14 | 67.91±27.86 | 14 | 64.85±21.03 | 14 | 62.46±20.14 | 12 | 65.05±26.68 |
| ptc=1 | 28 | 52.78±15.40 | 30 | 51.88±16.95 | 29 | 51.75±17.79 | 25 | 53.28±15.11 |
| ptc≥2 | 21 | 46.09±20.00 | 19 | 44.78±20.20 | 17 | 43.18±23.73 | 12 | 42.84±22.70 |

**Table S4.** The disparity in renal function post-transplantation among ABMR patients with different ptc scores

| ANOVA | 1-year after transplantation | | 2-year after transplantation | 3-year after transplantation | 5-year after transplantation |
| --- | --- | --- | --- | --- | --- |
| F value | 4.979 | 4.593 | | 3.517 | 3.595 |
| *P* value | 0.010 | 0.014 | | 0.036 | 0.035 |
| R^2^ | 0.142 | 0.133 | | 0.110 | 0.135 |

**Table S5.** The disparity in renal function post-transplantation among ABMR patients was compared based on different ptc scores.

|  | Tukey’s multiple comparison | Mean difference | 95% confidence interval, 95% CI | Adjusted *P* value |
| --- | --- | --- | --- | --- |
|  | ptc=0 vs ptc=1 | 15.13 | (-0.76,31.02) | 0.065 |
| 1-year after transplantation | ptc=0 vs ptc≥2 | 21.82 | (5.07,38.57) | 0.008 |
|  | ptc=1 vs ptc≥2 | 6.69 | (-7.32,20.71) | 0.489 |
|  | ptc=0 vs ptc=1 | 12.97 | (-1.73,27.67) | 0.094 |
| 2-year after transplantation | ptc=0 vs ptc≥2 | 20.07 | (4.08,36.07) | 0.010 |
|  | ptc=1 vs ptc≥2 | 7.10 | (-6.21,20.42) | 0.411 |
|  | ptc=0 vs ptc=1 | 10.71 | (-5.07,26.49) | 0.240 |
| 3-year after transplantation | ptc=0 vs ptc≥2 | 19.29 | (1.79,36.79) | 0.028 |
|  | ptc=1 vs ptc≥2 | 8.58 | (-6.24,23.39) | 0.351 |
|  | ptc=0 vs ptc=1 | 11.77 | (-5.51,29.04) | 0.235 |
| 5-year after transplantation | ptc=0 vs ptc≥2 | 22.21 | (2.13,42.29) | 0.027 |
|  | ptc=1 vs ptc≥2 | 10.44 | (-6.83,27.72) | 0.317 |

**Table S6.** Banff score for patients.

|  | Total | ABMR | TCMR | STA | *P* value |
| --- | --- | --- | --- | --- | --- |
| i | 1±0 | 1±0 | 1±0 | 1±0 | - |
| t | 0.5±0.54 | 0.5±0.71 | 1±0 | 0 | 0.031 |
| v | 0.5±0.54 | 0.5±0.71 | 1±0 | 0 | 0.031 |
| ptc | 1.13±1.13 | 2.5±0.71 | 1±1 | 0.33±0.58 | 0.076 |
| g | 1±0.76 | 2±0 | 1±0 | 0.33±0.58 | 0.011 |
| C4d+ | 0.13±0.35 | 0 | 0.33±0.58 | 0 | 0.507 |
| cg | 0 | 0 | 0 | 0 | - |
| ci | 0.25±0.46 | 1±0 | 0 | 0 | - |
| ct | 0.25±0.46 | 1±0 | 0 | 0 | - |
| cv | 0.13±0.35 | 0.5±0.71 | 0 | 0 | 0.247 |
| ah | 0.38±0.74 | 1±1.41 | 0.33±0.58 | 0 | 0.393 |
| mm | 0.25±0.46 | 1±0 | 0 | 0 | - |
| ti | 0.88±0.35 | 1±0 | 1±0 | 0.67±0.58 | 0.507 |
